# The Effects of Extracorporeal Magnetic Innervation in the Treatment of Women with Urinary Incontinence: A Systematic Review

**DOI:** 10.3390/jcm12175455

**Published:** 2023-08-22

**Authors:** Katarzyna Strojek, Agnieszka Strączyńska, Agnieszka Radzimińska, Magdalena Weber-Rajek

**Affiliations:** Department of Physiotherapy, Nicolaus Copernicus University Collegium Medicum in Bydgoszcz, 85-067 Bydgoszcz, Poland; radziminska@cm.umk.pl (A.R.); m.weber@cm.umk.pl (M.W.-R.)

**Keywords:** extracorporeal magnetic innervation, urinary incontinence, women

## Abstract

Purpose: The aim of this study is to identify and critically evaluate literature regarding the clinical efficacy of extracorporeal magnetic innervation (ExMI) in the treatment of female patients with urinary incontinence (UI). Methods: An analysis was carried out using the following electronic databases: Medline, PubMed, ScienceDirect, and the Cochrane Library (data published between 2008 and 2023). Searches of the above databases were conducted in April 2023. Only randomized clinical studies (RCTs) in English studies were eligible for the study. Randomized controlled trials were included in the review and evaluated with the Downs and Black checklist. Results: Eleven studies met the inclusion criteria. Among these, two studies examined the use of ExMI and PMFT (pelvic floor muscle training) and three studies compared active ExMI versus sham ExMI. Four studies evaluated solely ExMI, and moreover, there was no control group in two of these studies. One study compared the effects of Kegel exercises with ExMI, while another study compared electrostimulation with ExMI. The reviewed studies exhibited significant differences in interventions, populations, and outcome measures. Conclusions: Extracorporeal magnetic stimulation has shown promise as an effective treatment for female urinary incontinence. Whether used alone or as a component of combination therapy, ExMI has the potential to enhance patients’ quality of life (QoL) without significant safety concerns.

## 1. Introduction

Extracorporeal magnetic innervation is a non-invasive, non-surgical therapy utilized in the conservative treatment of urinary incontinence, including stress, urgency, and mixed types. ExMI involves stimulation of the pelvic floor muscles through the induction of an electric current with the aid of a magnetic field [1]. It is believed that ExMI produces similar effects to functional electrical stimulation by generating impulses with a steep magnetic field slope that can penetrate various substances, such as air and human tissues, without attenuation. The time-varying magnetic field induces electrical potential, resulting in ion flows or eddy currents within the tissues. These ion flows depolarize resting membrane potentials of the motor neurons in the celiac and pudendal nerves.

Subsequently, the initiation and propagation of an action potential occur along the axon as a result of the flow of Na+ and K+ ions. The impulses reaching the motor plates elicit activity in the pelvic floor muscles, which consequently contract and relax in response to each impulse [2]. ExMI™ technology [3] strengthens all layers of the pelvic floor muscles, aids in the rebuilding of strength and endurance, and also facilitates one’s control over urination and bowel movements. Additionally, ExMI improves circulation in the pelvic region. Ismail et al. [4] reported that 52.1% of their female patients experienced side effects from ExMI such as leg, abdominal, and back pain; cystitis; bowel symptoms; as well as tingling sensations [1]. Moreover, the use of ExMI is also associated with a limited number of contraindications—pregnancy, the presence of an intrauterine contraceptive device, a hip prosthesis, a pacemaker, and severe arrhythmias [5].

In 2013, the Fifth International Consultation on Incontinence [6] emphasized the lack of sufficient scientific evidence to make recommendations regarding the use of ExMI. Furthermore, it was highlighted that further research is crucial to reduce the uncertainty surrounding decisions concerning ExMI implementation. Numerous key aspects remain unknown, such as the efficacy of ExMI for UI in women, the optimal number of treatment sessions required, and the duration of its effects. Without a doubt, addressing these queries requires conducting and accessing high-quality randomized clinical trials involving ExMI. Considering the limited number of scientific reports available which would clearly determine the effectiveness of ExMI in treating UI, our paper aims to comprehensively analyze the existing clinical trials.

## 2. Methods of Identification of Studies

A comprehensive analysis of the available literature was performed in order to obtain the relevant clinical data needed with the aim of creating the following systematic review. The analysis was carried out in the following electronic databases: Medline, PubMed, ScienceDirect, and the Cochrane Library (data from the years 2008 to 2023). The authors searched these databases in April 2023. Literature search strategies were developed using medical subject headings for extracorporeal magnetic innervation, as well as text words associated with urinary incontinence in women. The search terms included following clinical keywords: “extracorporeal magnetic innervation” OR “magnetic stimulation” AND/OR “urinary incontinence” AND/OR “women”.

### 2.1. Study Selection

The researchers’ initial selection involved reviewing titles and abstracts. Studies that failed to meet the eligibility criteria were excluded at this stage. Each article was independently reviewed by two reviewers (K.S. and A.S.), and any discrepancies between them were resolved through discussion. Additionally, duplicate citations resulting from database overlap were excluded. Subsequently, full texts of all articles that potentially met the inclusion criteria were retrieved. Studies irrelevant to extracorporeal magnetic innervation or lacked clear applicability to female urinary incontinence were not included. The researchers also excluded studies that did not mention the terms “magnetic stimulation” or “urinary incontinence” in the text. Studies that described the use of ExMI in other disorders or diseases other than UI or that were conducted exclusively in male subjects, were also excluded. Additionally, all original research articles and randomized clinical trials were included in the study. The literature search was limited to studies conducted in the English and involving human subjects.

Furthermore, we used the PRISMA (Preferred Reporting Items for Systematic Reviews and Meta-Analyses) flow diagram to illustrate the selection process of relevant publications [7]. Methodological quality of the selected studies was assessed using the Downs and Black questionnaire, which is a widely utilized tool in evaluating the methodological quality of controlled trials [8].

### 2.2. Data Extraction

For each article included in this analysis, the following data were extracted: type and location of the study, study objective, author’s name, year of publication, participants’ information (number, age, and type of UI), intervention type and duration, study design, and key findings.

### 2.3. Quality Assessment

The methodological quality of the trials was assessed using the Downs and Black checklist [8], adapted from Hartling and Hignett [9,10]. The questionnaire consisted of 27 questions and included 4 assessment categories for evaluation: reporting, external validity, internal validity/bias, and internal validity/confounding (Table 1). Each eligible article was assessed using this scoring system and was subsequently classified as high, moderate, limited, or poor quality. The Downs and Black questionnaire was found to be valid and reliable for the critical evaluation of experimental and non-experimental research. Two evaluators (K.S. and A.S.) independently classified the data using the checklist. 

## 3. Results

**Figure 1:**
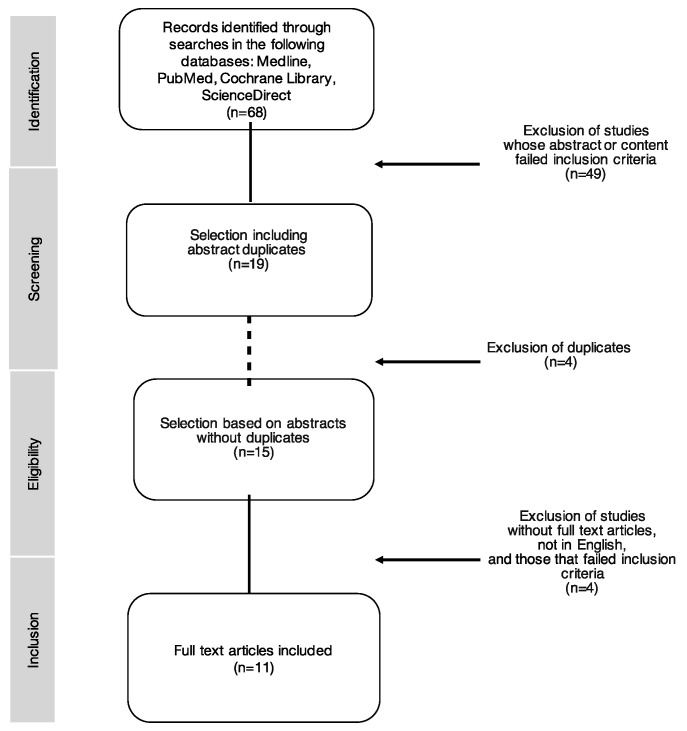
Process of study selection according to the PRISMA flow diagram.

The literature search yielded 68 papers. Following the initial selection process, involving screening titles and abstracts, 19 studies were left. The 49 remaining studies did not meet the preliminary criteria. At the second selection phase, four duplicates were excluded from the initial 19 papers. During the final selection phase, where the authors examined full-text versions of the studies, 4 more studies were excluded, thus resulting in 11 studies that met the inclusion criteria for the present review. The flow of studies through the selection process is shown in Figure 1.

### 3.1. Study Details

Upon entering the keywords, 68 studies were found in the databases. Among these, 49 did not meet the inclusion criteria and an additional 4 articles were duplicates. As a result, 11 manuscripts that met the eligibility criteria were included in the analysis (Table 2). Out of the eleven studies, eight were randomized controlled trials [11,12,13,14,15,16,17], while the other three were RCTs that employed a crossover design where patients acted as their own control group [18,19,20]. All studies reported data both before and after the intervention. Table 2 summarizes the important characteristics of the included studies. The main objective of the selected studies was to evaluate the use of ExMI in the treatment of urinary incontinence.

### 3.2. Outcomes Reported

Eleven articles were included in the final review (Table 3). There was a total of 943 patients (EG = 554, CG = 524) aged 45–75 in all study groups. While one study enrolled patients over 21 years of age, the exact mean age of the participants was not specified [13]. Among the qualified studies, four originated from Türkiye, two from Poland, and one each from New Zealand, Australia, Japan, Malaysia, and Croatia [11,12,13,14,15,16,17,18,19,20,21].

The study population ranged from 13 to 151 patients. In the 11 analyzed studies, women with stress urinary incontinence (SUI) were included in the study groups, with one study also including women with urgency urinary incontinence (UUI) [12]. In another manuscript, both women with SUI and UUI were included in the study [18]. Out of the 11 studies that met the inclusion criteria, 2 examined the use of ExMI in combination with PMFT (pelvic floor muscle training) [15,21]. Three papers compared active ExMI with sham ExMI [11,12,13]. In three studies, ExMI alone was assessed [14,18,19,20], with two of the three studies lacking a control group [18,19,20]. Additionally, one study compared the effects of Kegel exercises with ExMI [17], and another—electrostimulation with ExMI [16]. Significant differences were observed in terms of interventions, study populations, and outcome measures across the reviewed studies.

The intervention duration spanned 4–8 weeks, with the majority of procedures lasting for 6 weeks. Treatment sessions were conducted 2–5 times a week, lasting between 15 to 30 min each. Scientific studies covered in this review employed various diagnostic tests, such as urodynamic tests, a bladder diary, a pad test, and myostatin concentration. The strength and function of pelvic floor muscles were also tested using instruments such as a perineometer, a circumvaginal muscle rating score, and electromyography. Moreover, each selected study evaluated the quality of life of patients before and after therapy using reliable and standardized assessment tools, such as the Urinary Incontinence Quality of Life Scale for female patients (I-QOL), the King’s Health Questionnaire (KHQ), the Urogenital Distress Inventory (UDI-6), the International Consultation on Incontinence Questionnaire—Urinary Incontinence Short Form (ICIQ-UI SF), the International Prostate Symptom Score-QoL Index (IPSS-QoL), the International Consultation on Incontinence Questionnaire Lower Urinary Tract Symptoms Quality of Life Module (ICIQ-LUTS-QoL), Patient Global Impression of Improvement scale (PGI-I), the Revised Urinary Incontinence Scale (RUIS), the General Self-Efficacy Scale (GSES), and the Beck Depression Inventory scale (BDI-II) [11,12,13,14,15,16,17,18,19,20,21]. 

The results of the analyzed studies indicate that following ExMI treatment sessions, urinary symptoms and incontinence conditions decreased [11,14,18,19,20]. The research proved that PFMT and ExMI are effective treatment methods for SUI in women [15,21] and demonstrated higher patient satisfaction with ExMI treatment compared to Kegel exercises [17].

### 3.3. Critical Appraisal

All studies were analyzed using the Downs and Black checklist, which allows for accurate assessment of both comparative and non-comparative studies (Table 4). The findings of this systematic review demonstrate moderate methodological quality across the included studies, with a mean score of 17 out of 27. Subsequently, there is variability in the data that stems from differences in study design. The highest critical score, averaging 22 out of 27, was attributed to studies with a well-described randomization, while studies with control groups achieved a score of 16 out of 27.

Four RTCs demonstrated high methodological quality, scoring 22 out of 27 [13,14,15,17]. They showed relative consistency across most checklist categories. These studies reported information on the number and means of double blinding, included a control group, and had sufficient statistical power to detect clinically significant effects. The analysis of research quality revealed that the publication date of the studies influenced the number of parameters met in the Downs and Black checklist.

Publications from 2008 to 2014 showed research methodology ranging from 44% to 74%, indicating limited or moderate quality levels [11,12,18,19,20,21]. Nevertheless, studies published after 2014 achieved results ranging from 52% to 81%, indicating moderate or strong quality indices [13,14,15,16,17]. Overall, the analyzed articles demonstrate a high degree of heterogeneity in terms of research methodology.

## 4. Discussion

The objective of the study was to evaluate and analyze the effectiveness of extracorporeal magnetic stimulation in the treatment of urinary incontinence in women. In view of the obtained results, improvements in UI were observed after ExMI treatment, regardless of the type of UI. Moreover, most studies demonstrated the effectiveness of ExMI primarily in stress urinary incontinence. In recent years, ExMI has mainly been compared to placebo or controlled trials [22]. Despite the fact that these studies demonstrated the superiority of ExMI in treating SUI with a low incidence of side effects, there is still a lack of studies evaluating the pharmacoeconomic impact of conservative methods of SUI treatment. The authors of this paper believe that this aspect should be investigated in future research.

There is still a lack of studies that standardize the use of ExMI in terms of dosage, duration of the procedure, or period of application. There are single scientific reports that specify the side effects, effectiveness, or evaluation of ExMI as a standalone or a component of combination therapy. In a study by Ismail et al. [4], 52.1% of patients withdrew from ExMI therapy, while 35.4% experienced side effects. Yamanishi et al. [12] confirmed the effectiveness of magnetic stimulation in the treatment of an overactive bladder. Hoscan et al. [23] and Galloway et al. [1,2] showed the very beneficial effects of this therapy in the treatment of mixed form and stress urinary incontinence. Meanwhile, a study by Gumussoy et al. [16] did not detect any statistically significant differences between all measured variables for biofeedback-guided pelvic floor muscle training (EMG-BF), with or without ExMI at the initial and final assessments. The authors found no additional benefit to dual therapy. 

This could signify that the high quality of the randomized study could be attributed to several factors, such as the presence of a sham group, an estimation of the number of trials, the testing of a methodology with reliable outcome indicators, as well as long-term follow-up [24].

Most studies were of average quality, although more recent studies were of better quality in terms of both randomization and blinding. Confidence intervals tended to be wide, except in more recent studies, and it is still difficult to reliably identify or rule out the useful effects of available ExMI studies. Additional clinical research should be performed to evaluate the impact of ExMI on the function of pelvic floor muscles in women with urinary incontinence, because the optimal parameters and proper and precise methodology of this procedure have yet to be established.

## 5. Conclusions

Extracorporeal magnetic stimulation may be effective in treating female urinary incontinence. Whether applied as a standalone treatment or a component of combination therapy, it can improve the quality of life of patients without major safety concerns. Nevertheless, researchers should draw conclusions from the findings with extreme care due to the high degree of heterogeneity in terms of the quality and reliability of the methodology in the available studies. Further in-depth research is needed to evaluate the long-term efficacy of this promising alternative treatment for urinary incontinence.

As the emphasis on methodology for conducting clinical trials continues to increase, the available studies on the use of extracorporeal magnetic stimulation in female urinary incontinence are gaining credibility.

## Figures and Tables

**Table 1:** The categorization of total scores of the Downs and Black checklist, adapted from Hartling and Hignett.

Quality Index	Percentage	Methodological Quality Score (*n* = 27)
Strong	≥75%	≥21
Moderate	50–74%	14–21
Limited	25–49%	7–13
Poor	<25%	<7

**Table 2:** Characteristics of the included studies.

Study	Design	Study Objective
Giling et al., 2008 [11]	RCT	Compare the efficacy of ExMI of the pelvic floor in treating SUI vs sham ExMI
Doganay et al., 2010 [18]	RCT	Evaluate the long-term efficacy of ExMI in the treatment of women with UI
Bakar et al., 2011 [19]	RCT	Examine the effects of ExMI treatment on pelvic floor muscle strength, urinary symptoms, incontinence conditions, and QoL in older women with SUI
Hayes et al., 2012 [21]	RCT	Compare ExMI to standard PFMT supervised by an experienced pelvic floor physiotherapist for women with SUI
Akbayrak et al., 2014 [20]	RCT	Assess the effectiveness of MS in the treatment of SUI in women
Yamanishi et al., 2014 [12]	RCT	Evaluate the efficacy and safety of MS for the treatment of urinary incontinence and an overactive bladder
Lim et al., 2015 [13]	RCT	Present a protocol of a multicenter, randomized, double-blind, sham-controlled, parallel-group trial to evaluate the efficacy of MS for SUI
Weber-Rajek et al., 2018 [14]	RCT	Assess the effectiveness of ExMI in the treatment of SUI in women
Weber-Rajek et al., 2020 [15]	RCT	Assess the effectiveness of PFMT and ExMI in the treatment of UI in women with SUI
Gumussoy et al., 2021 [16]	RCT	Assess the effectiveness of EMG-BF, with and without ExMI therapy, on lower urinary tract symptoms, including the frequency of SUI and grams of urine loss, health-related quality of life, and sexual function in women with SUI
Mikuš et al., 2022 [17]	RCT	Estimate the effectiveness of Kegel exercises versus ExMI in the treatment of SUI

RCT—randomization clinical trials, ExMI—extracorporeal magnetic innervation, SUI—stress urinary incontinence, UI—urinary incontinence, QoL—quality of life, PFMT—pelvic floor muscle training, MS—magnetic stimulation, EMG-BF—biofeedback-guided pelvic floor muscle training.

**Table 3:** Basic information on design details and clinical outcomes of the included studies.

Study	Country	Assigned Group/ Sample Size/Type of UI	Type of Intervention	Intervention Duration/Number of Sessions	Intervention and Study Design	Results
Giling et al., 2008 [11]	New Zealand	N = 70 EG1 = 35 EG2 = 35 Age: 54.4 Type: SUI	EG1: active ExMI EG2: sham ExMI	6 Wks; 3 times × Wk, 20 min; patients have been educated on PMFT and encouraged to complete low-intensity PFMT at home	20 min of provocative pad test with a predetermined bladder volume, 3-day bladder diary, 24 h pad test, circumvaginal muscle rating score, I-QOL, KHQ	Results indicate that ExMI with low-intensity PFMT is no more effective than sham treatment with low-intensity PFMT
Doganay et al., 2010 [18]	Türkiye	N = 137 EG = 137 Age: 55.8 Type: SUI, UUI	EG: ExMI	8 Wks; 2 times × Wk, 20 min	Urodynamic testing, leakage number, 1 h pad test, I-QoL, VAS	All of the patients with UI were successfully followed up. Patients had a significant QOL and noticed a decrease in daily pad use and leakage episodes after treatment with ExMI
Bakar et al., 2011 [19]	Türkiye	N = 13 EG = 13 Age: 65.23 Type: SUI	EG: ExMI	6 Wks; 2 times × Wk, 20 min	Urinary symptoms, pelvic floor EMG activity, 1 h pad test, VAS, UDI-6, I-QoL	Urinary symptoms and incontinence conditions decreased after ExMI treatment sessions. The pad test results indicated a reduction in urine loss; scores of I-QoL, UDI-6 and VAS reduced after the treatment
Hayes et al., 2012 [21]	Australia	N= 65 EG1 = 33 EG2 = 32 Age: 50 Type: SUI	EG1: PFMT EG2: ExMI	EG1: 5 individual sessions EG2: 6 Wks, 2–3 times × Wk, 20 min	24 h pad test, ICIQ-UI SF,	At 3 months post-treatment, there appears to be no significant difference in treatment effects of PFMT and ExMI in women with SUI
Akbayrak et al., 2014 [20]	Türkiye	N = 20 EG = 20 Age: 47 Type: SUI	EG: ExMI	4 Wks; 5 times × Wk, 20 min	Pelvic floor EMG, 24 h pad test, UDI-6	After the treatment, there was a significant improvement in the amount of urinary leakage, the EMG activity of pelvic floor muscles, and UDI scores
Yamanishi et al., 2014 [12]	Japan	N = 151 EG1 = 94 EG2 = 49 Age: 66 Type: UUI	EG1: active ExMI EG2: sham ExMI	6 Wks; 2 times × Wk, 25 min	Number of leakage episodes, IPSS-QoL	MS is effective for the treatment of UUI in women with an overactive bladder
Lim et al., 2015 [13]	Malaysia	N = 120 EG1 = 60 EG2 = 60 Age ≥ 21 Type: SUI	EG1: active ExMI EG2: sham ExMI	8 Wks; 2 times × Wk, 20 min	Incontinence episode diary, 1 h pad test, ICIQ-UI-SF, PGI-I, CIQ-LUTS-QoL, EQ-5D	MS is effective for SUI
Weber-Rajek et al., 2018 [14]	Poland	N = 52 EG = 28 CG = 24 Age: 65.41 Type: SUI	EG: ExMI CG: No intervention	4 Wks; 3 times × Wk, 15 min	RUIS, BDI-II, myostatin concentration, GSES	A statistically significant improvement in severity of UI and depression severity and a decrease in myostatin concentration
Weber-Rajek et al., 2020 [15]	Poland	N = 128 EG1 = 44 EG2 = 44 CG = 40 Age: 68.77 Type: SUI	EG1: PFMT EG2: ExMI EG3: No intervention	EG1: 4 Wks; 3 times × Wk, 45 min, supervised PMFT EG2: 4 Wks; 3 times × Wk, 15 min	RUIS, BDI-II, GSES, KHQ	PFMT and ExMI proved to be effective treatment methods for SUI in women
Gumussoy et al., 2021 [16]	Türkiye	N = 70 EG1 = 35 EG2 = 35 Age: 50.9 Type: SUI	EG1: EMG-BF EG2: EMG BF and ExMI	EG1: 8 Wks; 2 times × Wk, 20 min, and PFMT exercises at home EG2: 8 Wks; 2 times × Wk, 20 min, and PFMT exercises at home ExMI 6 Wks; 2 times × Wk, 20 min	1 h pad test, 3-day bladder diary, I-QOL, FSFI, PFMF, MOS	The authors did not find any statistically significant differences between all measured variables for the EG1 and EG2 at the initial and final assessments. The authors found no additional benefits to dual therapy
Mikuš et al., 2022 [17]	Croatia	N = 117 EG1 = 48 EG2 = 46 Age: 48.33 Type: SUI	EG1: Kegel exercises EG2: ExMI	EG1: 8 Wks; Kegel exercises at home EG2: ExMI 8 Wks, 2 × Wk, 30 min	PFMF, PGI-I scale, ICIQ-UI-SF, ICIQ-LUTS-QoL	Patients treated with ExMI had a lower number of incontinence episodes, a better QoL, and higher overall satisfaction with treatment than patients who performed Kegel exercises

UI—urinary incontinence, N—number of participants, UUI—urgency urinary incontinence, SUI—stress urinary incontinence, EG—experimental group, ExMI—extracorporeal magnetic innervation, Wks—weeks, min—minutes, Wk—week, PFMT—pelvic floor muscle training, I-QOL—Urinary Incontinence Quality of Life Scale for female patients, KHQ—King’s Health Questionnaire, VAS—visual analog scale, UDI-6—Urogenital Distress Inventory, EMG—electromyographic activity, ICIQ-UI SF—International Consultation on Incontinence Questionnaire—Urinary Incontinence Short Form, IPSS-QoL—International Prostate Symptom Score-QoL Index, MS—magnetic stimulation, ICIQ-LUTS-QoL—International Consultation on Incontinence Questionnaire Lower Urinary Tract Symptoms Quality of Life Module, PGI-I—Patient Global Impression of Improvement scale, RUIS—Revised Urinary Incontinence Scale, GSES—General Self-Efficacy Scale, BDI-II—Beck Depression Inventory, EMG-BF—biofeedback-guided pelvic floor muscle training, PFMF—pelvic floor muscle function measured with a perineometer; MOS—Modified Oxford Scale, FSFI—Female Sexual Function Index.

**Table 4:** Quality assessment scores based on the modified Downs and Black checklist, adapted from Hartling and Hignett.

Study	Methodological Quality Score	Percentage	Quality Index
Giling et al., 2008 [11]	17	62%	Moderate
Doganay et al., 2010 [18]	13	49%	Limited
Bakar et al., 2011 [19]	12	44%	Limited
Hayes et al., 2012 [21]	13	49%	Limited
Akbayrak et al., 2014 [20]	9	33%	Limited
Yamanishi et al., 2014 [12]	20	74%	Moderate
Lim et al., 2015 [13]	22	81%	Strong
Weber-Rajek et al., 2018 [14]	22	81%	Strong
Weber-Rajek et al., 2020 [15]	22	81%	Strong
Gumussoy et al., 2021 [16]	14	52%	Moderate
Mikuš et al., 2022 [17]	22	81%	Strong

## Data Availability

Not applicable.
